# Outcomes of patients in Chagas disease of the central nervous system: a systematic review

**DOI:** 10.1017/S0031182023001117

**Published:** 2024-01

**Authors:** William J. Shelton, John M. Gonzalez

**Affiliations:** Grupo de Ciencias Básicas Medicas, School of Medicine, Universidad de Los Andes, Bogotá, Colombia

**Keywords:** central nervous system, cerebral Chagas, Chagas disease, protozoan infections

## Abstract

Chagas disease is a parasitic infection caused by the protozoan *Trypanosoma cruzi*. One of the complications of the disease is the infection of the central nervous system (CNS), as it can result from either the acute phase or by reactivation during the chronic phase, exhibiting high mortality in immunocompromised patients. This systematic review aimed to determine clinical and paraclinical characteristics of patients with Chagas disease in the CNS. Articles were searched from PubMed, Scopus and LILACS until January 2023. From 2325 articles, 59 case reports and 13 case series of patients with Chagas in the CNS were retrieved from which 138 patients were identified. In this population, 77% of the patients were male, with a median age of 35 years old, from which most of them came from Argentina and Brazil. Most of the individuals were immunocompromised from which 89% were HIV-positive, and 54 patients had an average of 48 cells per mm^3^ CD4+ T cells. Motor deficits and seizures were the most common manifestation of CNS compromise. Furthermore, 90 patients had a documented CNS lesion by imaging from which 89% were supratentorial and 86% were in the anterior/middle cranial fossa. The overall mortality was of 74%. Among patients who were empirically treated with anti-toxoplasma drugs, 70% died. This review shows how Chagas disease in the CNS is a devastating complication requiring prompt diagnosis and treatment to improve patients’ outcomes.

## Graphical abstract

**Figure:**
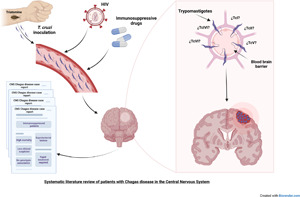

## Introduction

*Trypanosoma cruzi* is the pathogen that causes Chagas disease, a neglected tropical disease with high impact on populations with poor socioeconomic status, particularly in 21 Latin American countries (Brenière *et al*., 2016; Pérez-Molina and Molina, 2018). *Trypanosoma cruzi* is classified into 7 genotypes known as discrete typing units or DTUs (Tibayrenc and Ayala, 2022; Zingales and Bartholomeu, 2022). This molecular typing could help in differentiating characteristics such as tissue and organ involvement, among other variables associated with Chagas pathogenesis such as the parasite load and the immune response (Brenière *et al*., 2016; Medina-Rincón *et al*., 2021).

The disease is divided into an acute and a chronic phase. The acute phase could manifest as unspecific symptoms and usually lasts 4–8 weeks. All patients who become infected with *T. cruzi* enter an early chronic phase, termed as asymptomatic or indeterminate. Approximately 20–40% of infected individuals progress to a symptomatic chronic phase, primarily characterized by organ involvement, with cardiomyopathy being the most common, followed by the development of megavisceral manifestations, such as megaoesophagus and megacolon (Bern, 2015; Pérez-Molina and Molina, 2018). One of the complications of Chagas disease is the presence of the parasite in the central nervous system (CNS) in the acute phase or due to reactivation during chronic disease (Useche *et al*., 2022). This reactivation is particularly prevalent in immunocompromised patients (Cordova *et al*., 2008; Clark and Bern, 2021; Useche *et al*., 2022). CNS Chagas disease exhibits varying mortality rates depending on the disease phase, with mortality near 10% in the acute disease and 79–100% during reactivation (Pittella, 2009; Pérez-Molina *et al*., 2011). Common clinical signs and symptoms observed in patients with cerebral Chagas include confusion, headache, hypertonia, convulsions, meningismus, focal neurological deficits, fever and altered mental status (Pittella, 1993; Córdova *et al*., 2013). The diagnosis of cerebral Chagas disease is challenging, typically involves direct observation of parasitic forms in the cerebrospinal fluid (CSF), blood or tissue biopsies (Córdova *et al*., 2013; Useche *et al*., 2022). Chagas disease reactivation in the CNS can manifest as acute diffuse meningoencephalitis or as a mass-occupying abscess-like lesion (chagoma) in 75–90% of the patients (Cordova *et al*., 2008; Córdova *et al*., 2013). Typically, neuroimaging reveals abnormalities, with common findings of single supratentorial abscess-like lesions (Cordova *et al*., 2008). However, some presentations are atypical, with reports indicating cerebral tumour-like lesions or involvement in rare anatomical locations, such as in the suprasellar region (Choi *et al*., 2004; Sica *et al*., 2008). It is worth noting that neither neuroimaging modalities nor clinical symptoms can reliably distinguish chagasic manifestations in the CNS from those caused by more prevalent pathogens or non-infectious diseases. Nevertheless, normal neuroimaging does not necessarily rule out CNS involvement (Cordova *et al*., 2008). A high clinical suspicion of Chagas disease is required to reduce the mortality in these patients (DiazGranados *et al*., 2009; Clark and Bern, 2021).

The aim of this study is to conduct a systematic review of the literature to determine whether there is a pattern of Chagas disease, specifically regarding clinical and paraclinical outcomes of patients with Chagas disease affecting the CNS.

## Materials and methods

### Literature search and strategy

Three databases were reviewed: PubMed (Medline), Scopus and Lilacs. The PRISMA guidelines for systematic reviews and meta-analyses (http://www.prisma-statement.org/) were used as the criteria for the literature analysis. This systematic review is currently under review on PROSPERO with registration number CRD42023446269. For PubMed, the following search terms were used: (((human[MeSH Terms]) AND ((*Trypanosoma cruzi*[MeSH Terms]) OR (chagas disease[MeSH Terms]) OR (trypanosomiasis[MeSH Terms])) AND ((Brain[MeSH Terms]) OR (Central Nervous System[MeSH Terms]) OR (Nervous System[MeSH Terms]) OR (Central Nervous System Protozoal Infections[MeSH Terms]) OR ‘Central Nervous System Parasitic Infections’[Mesh]) OR (Central Nervous System Diseases/parasitology[Mesh])) AND (Case reports[Publication Type])); for Scopus: (Human AND (‘Chagas disease’ OR ‘*Trypanosoma cruzi*’) AND ‘Infection’ AND ‘Central Nervous System’) ALL (‘Case Report’ OR ‘Case Series’); and for Lilacs (in Spanish): (Enfermedad de Chagas) OR (*Trypanosoma cruzi*) OR (Tripanosomiasis) AND (Sistema nervioso central) OR (Sistema nervioso) OR (Cerebro) AND (Infección).

### Study selection and ethics statement

This review will focus on observational studies, including only case reports and case series. Two reviewers screened independently titles and abstracts of all retrieved articles. Rayyan (https://www.rayyan.ai/) was used to identify duplicated articles. Each reviewer made the pertinent exclusions and inclusions in an independent manner. Discrepancies were resolved by consensus. This project was performed in accordance with the ethics committee of Universidad de Los Andes (October 2020).

### Inclusion/exclusion criteria

There were included case reports or case series available in literature reported between 1990 and 2023. It only included studies that meet the CARE guidelines (https://www.care-statement.org/) and Joanna Briggs Institute Critical Appraisal Checklist for Case reports and for Case series (https://jbi.global/critical-appraisal-tools). Additionally, studies that fulfilled the following inclusion criteria were incorporated: (i) patients with a history of documented Chagas disease (*T. cruzi*) in the CNS, confirmed by direct observation of parasitic forms in the CSF, brain biopsy or blood smear, along with clinical or imaging findings compatible with CNS infection, or by direct molecular methods (polymerase chain reaction) from CSF/brain biopsy used to diagnose the parasite in the CNS; (ii) patients with basic sociodemographic and clinical characteristics reported; (iii) articles identified as case reports or case series describing patient presentations and (iv) articles written in English or Spanish with an abstract in either language. Case reports were excluded if they consisted of (i) patients with congenital Chagas disease, (ii) reports without demonstration of CNS *T. cruzi* infection, (iii) studies without any case descriptions and (iv) abstracts or articles that were not available in full text.

### Data extraction of the selected articles

The following data were extracted into a data collection sheet: sociodemographic and clinical/characteristics (age, gender, country and clinical manifestations), imaging tests, laboratory findings (including reported DTUs), treatments used and mortality. If data were not mentioned in the article, it was assumed to be not reported. We reviewed articles published up to January 2023, see Supplementary Table 1.

### Data presentation and approximation

The collected data were presented using descriptive statistics for both qualitative and quantitative values. For quantitative data, a normality test (Shapiro–Wilk) was applied to determine the distribution as either parametric or non-parametric. Based on these results, non-parametric values were presented as median with interquartile range (IQR) (25^th^–75th percentile), while parametric values were presented as mean with standard deviation. GraphPad Prism V. 9.5.1 was used for descriptive and statistical analysis.

## Results

### Literature search results

**Figure 1.**
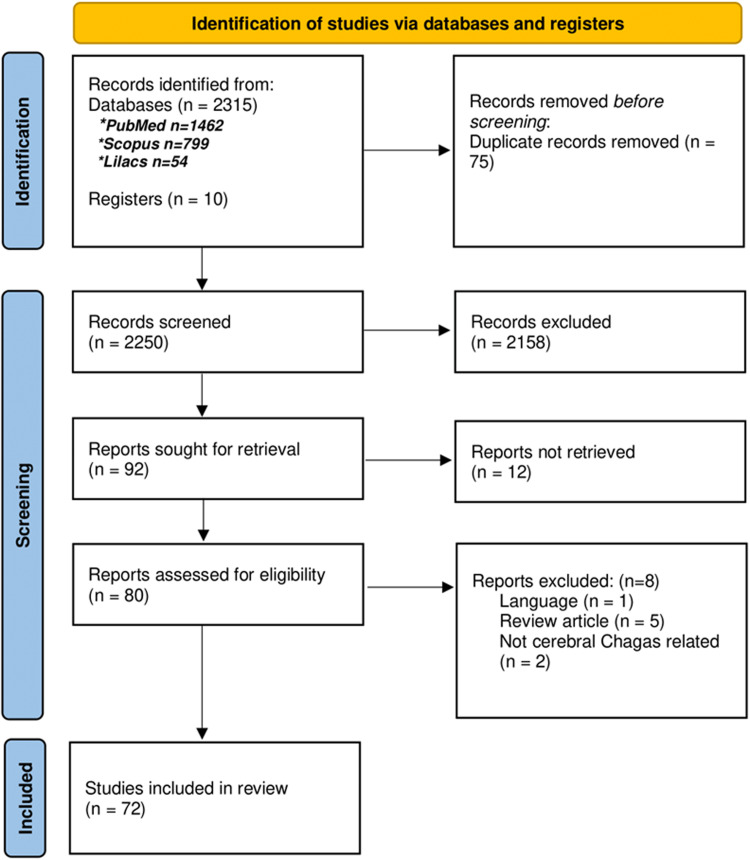
Flowchart of the systematic review realized following PRISMA guidelines for systematic reviews.

In the initial search, 2325 articles were identified: 2315 articles from the databases and 10 articles from additional manual searching. After removing duplicate articles (*n* = 75) 2250 articles were screened. Of those, 2158 articles did not meet the inclusion criteria, mainly because they were not case reports or case series or did not involve Chagas disease in the CNS. The remaining 92 articles were subjected to a full-text review, from which 12 could not be retrieved due to their year of publication and their lack of digitization. After the full-text review (*n* = 80), 8 articles were excluded; 2 case reports did not involve cerebral Chagas, 5 were review articles and 1 article was in a different language (exclusion criteria). In total, 72 articles met the final inclusion criteria for this systematic review (see Fig. 1 for PRISMA [Preferred Reporting Items for Systematic Reviews and Meta-Analyses] flowchart). Among the final articles chosen, 59 were case reports and 13 were case series.

### Characteristics of patients with Chagas disease in the central nervous system

**Figure 2.**
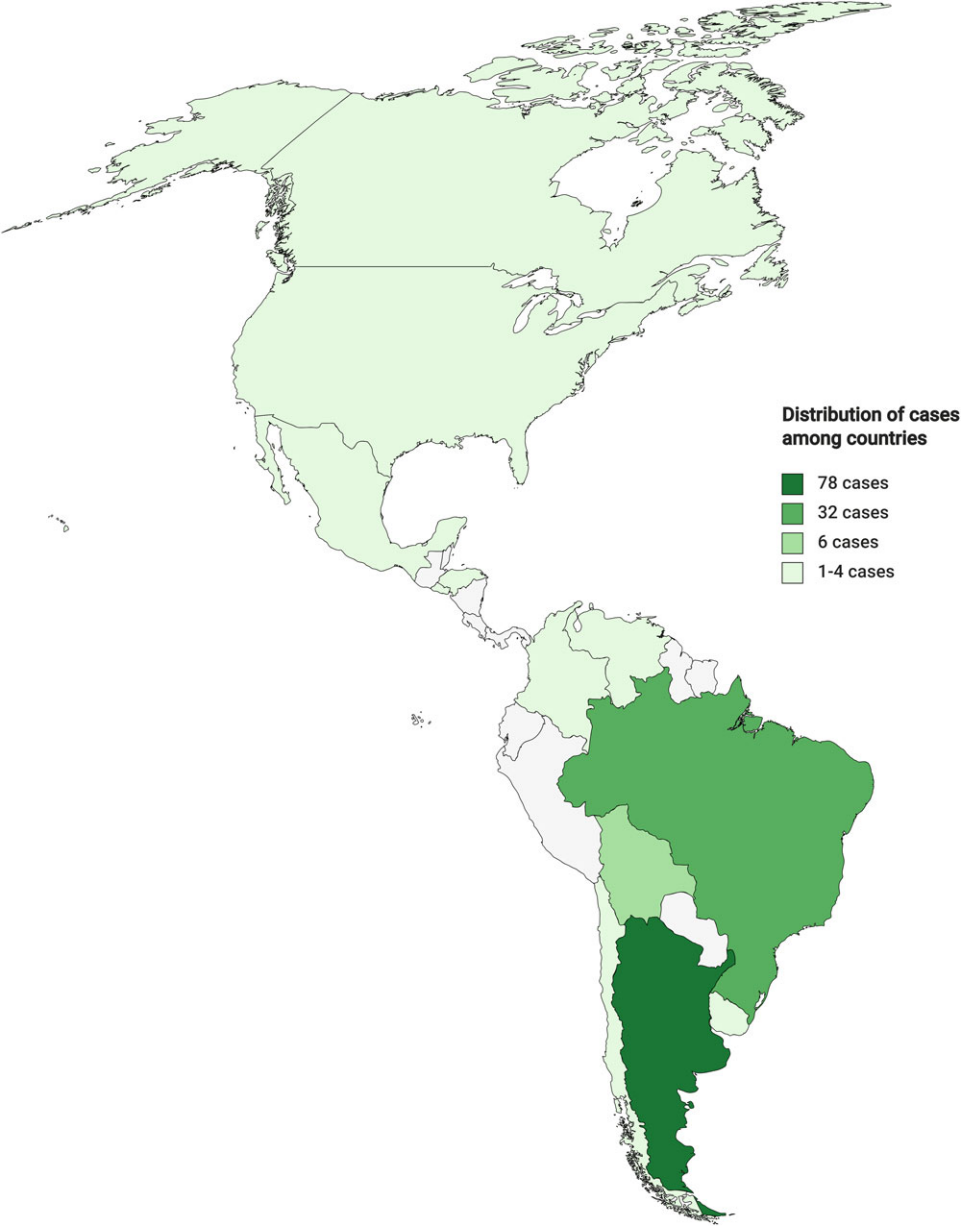
Country distribution of CNS Chagas patients. Colours represent the countries showing the number of cases obtained from the revised articles. This figure was created in: mapchart.net

In the 72 selected articles, there was a total of 138 patients. Information about individual age was available for 97 patients, with a median age of 35 years old (IQR^25–75^ 28–46.5). In this population, 77% of the patients were men (*n* = 106) while 19.5% were women (*n* = 27) and 3.6% (*n* = 5) did not report gender. According to the age, male patients had a median age of 35 years old (IQR 28–47), and female patients had a median age of 38 years old (IQR 32–49). Two series of articles with 24 and 15 patients reported age as a mean of 31 years old (IQR 22–52) and median of 33 years old (IQR 25–54), respectively. The most represented countries where patients came from were Argentina and Brazil, corresponding to 56 and 23% of the population, respectively (Fig. 2).

### Immunity status of reported patients with Chagas disease in the central nervous system

In this population, 98% of the patients (*n* = 135) were immunocompromised from which 89% were HIV-positive (*n* = 120). Additionally, there were 12 patients without HIV diagnosis with immunosuppression including 7 with organ transplant and under immunomodulators, 2 with leukaemia, 1 under chemotherapy, 1 receiving treatment for autoimmune disease and 1 patient with unclear cause. The remaining non-HIV patients (*n* = 3) were not proven to be immunocompromised. When evaluating the CD4+ T-cell counts, there were 54 patients out of 138 with lymphocytic values individually reported, in which the median was 48 cells per mm^3^ (IQR 18–102.5). The gender was documented in 50 out of 54 patients, from which 72% were male (*n* = 36) with a median of 46 cells per mm^3^ (IQR 18–101.5) and 28% were female (*n* = 14) with a median of 37.5 cells per mm^3^ (IQR 14.2–85.2). When evaluating gender and CD4+ T-cell counts with Mann–Whitney, there was no significant difference (*P* = 0.54). In 2 case series, 15 patients had a CD4+ T-cell count of 64 cells per mm^3^ (IQR 1–240), and 12 out 24 reported values for 9 patients <100 cells per mm^3^; 2 patients 100–200 cells per mm^3^ and 1 patient with a CD4+ T-cell count of 270 cells per mm^3^.

### Neurological manifestations of Chagas disease in the central nervous system

**Figure 3.**
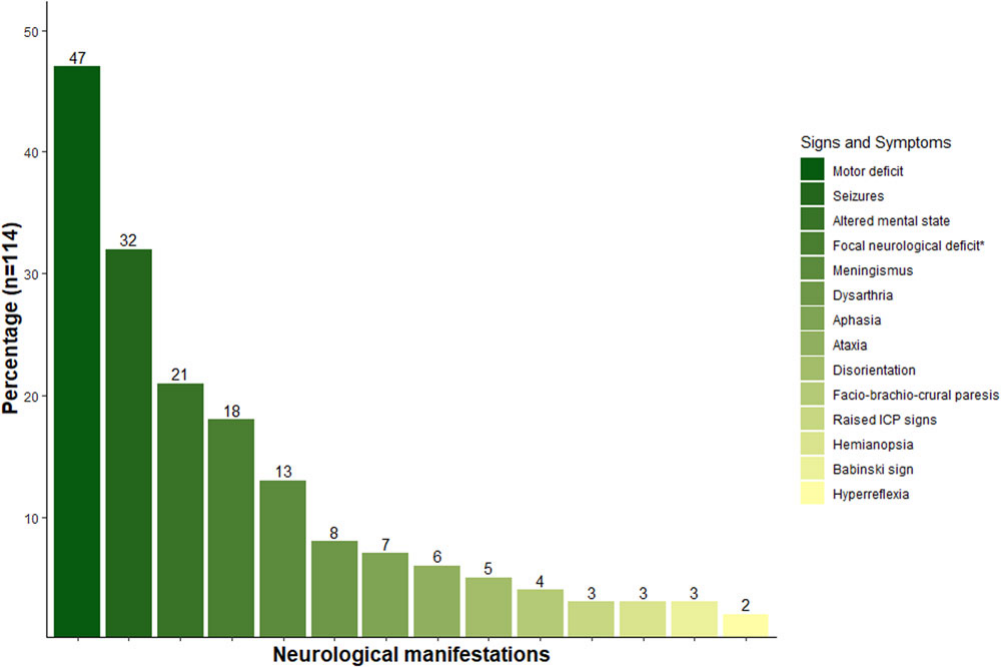
Neurological findings in patients with Chagas disease in the CNS. Bars represent the number of patients with the most common neurological signs and symptoms related to Chagas disease in the central nervous system. Other less common clinical findings are not described in this graph. *Focal neurological deficit not specified.

Physical examination was documented in 128 patients. Overall, it showed that 70% of patients (*n* = 90) presented a focal neurological sign (i.e. motor deficit) while 30% of the patients (*n* = 38) had no visible or observed neurological deficit. Patients that presented other clinical signs or symptoms (i.e. raised intracranial pressure signs) suggesting CNS involvement was seen in 89% of the population (*n* = 114, Fig. 3). From this group (*n* = 114), the most common symptom was motor deficit (47.3%), out of these, 4% (*n* = 2) debuted with paralysis of an extremity and 4% (*n* = 2) with paraplegia. The second most common finding was the presence of seizures (focal or generalized) in 32.4% of the population (*n* = 37). Other important variable was altered level of consciousness, in which 21% of the patients (*n* = 25) had a clinical deterioration of their state of consciousness. Cranial nerve deficits were found in 21% of the patients (*n* = 24) which were manifested as different clinical signs or symptoms (dysarthria, dysphagia, facial paralysis, hemianopia, anisocoria). Other least common symptoms such as meningeal irritation signs (i.e. neck stiffness) were present in 13.1% of the patients (*n* = 15). Cerebellar signs and symptoms were described in 10.5% of the population (*n* = 12). In terms of speech and language evaluation, 16% of cases (*n* = 18) had a deficit in which 9 patients had dysarthria, 8 had aphasia (not specified) and 1 had mutism (not specified). Only a minor proportion of the population presented upper motor neuron signs, having 7% (*n* = 8) of the patients with a clinical sign of pyramidal tract disfunction such as Babinski sign (*n* = 4), hyperreflexia (*n* = 3) and spasticity (*n* = 1). Two patients (*n* = 2) debuted with involuntary movements (hemichorea and myoclonus). Finally, the less common presentation were the abnormal sensorial modalities and frontal lobe syndromes, in which 3.5% of the population (*n* = 4) presented this.

### Systemic manifestations of Chagas disease in the central nervous system

**Figure 4.**
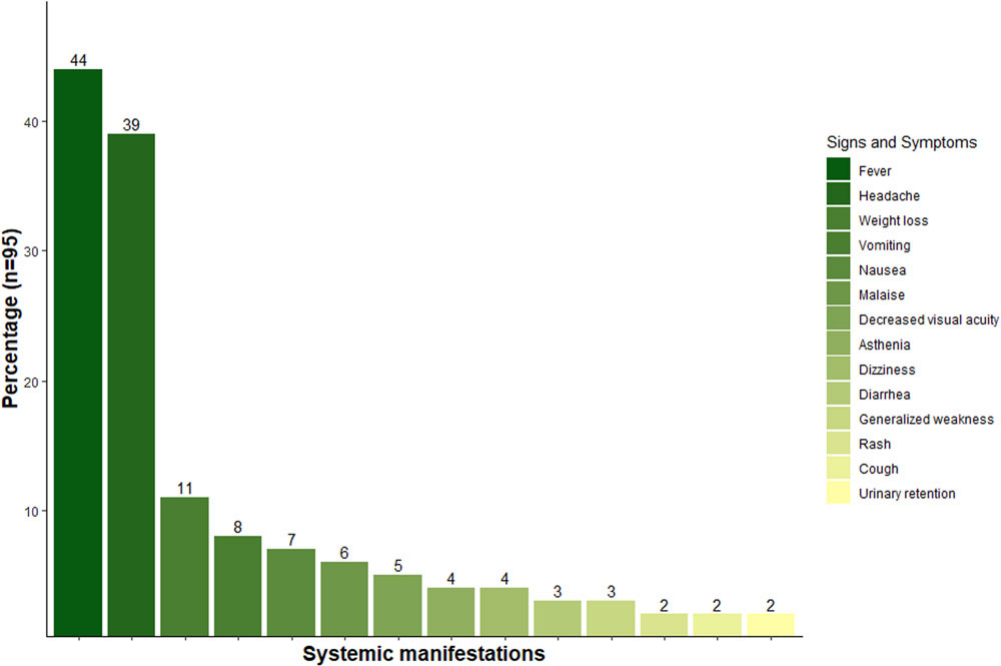
Systemic findings reported by patients with Chagas disease in the CNS. Bars represent the number of patients with different systemic manifestations during the diagnosis of Chagas disease in the central nervous system.

The included articles showed that out of 128 patients, 74.2% (*n* = 95) manifested either 1 or more systemic symptoms, while 25% (*n* = 33) did not report any symptom. Among the 95 symptomatic patients with CNS Chagas disease, the most prevalent symptom was fever and headache, affecting 83.1% of the patients (*n* = 79). Nausea and vomiting were reported in 16% of the patients (*n* = 15). Other findings included weight loss in 11.5% of the patients (*n* = 11) (Fig. 4). Additionally, various other symptoms and signs were present, such as malaise (*n* = 6), subjective decreased visual acuity (*n* = 5), asthenia (*n* = 4), dizziness (*n* = 4), diarrhoea (*n* = 4), generalized weakness (*n* = 3), rash (*n* = 2) and cough (*n* = 2).

### Anatomical location of Chagas lesions by imaging studies

**Figure 5.**
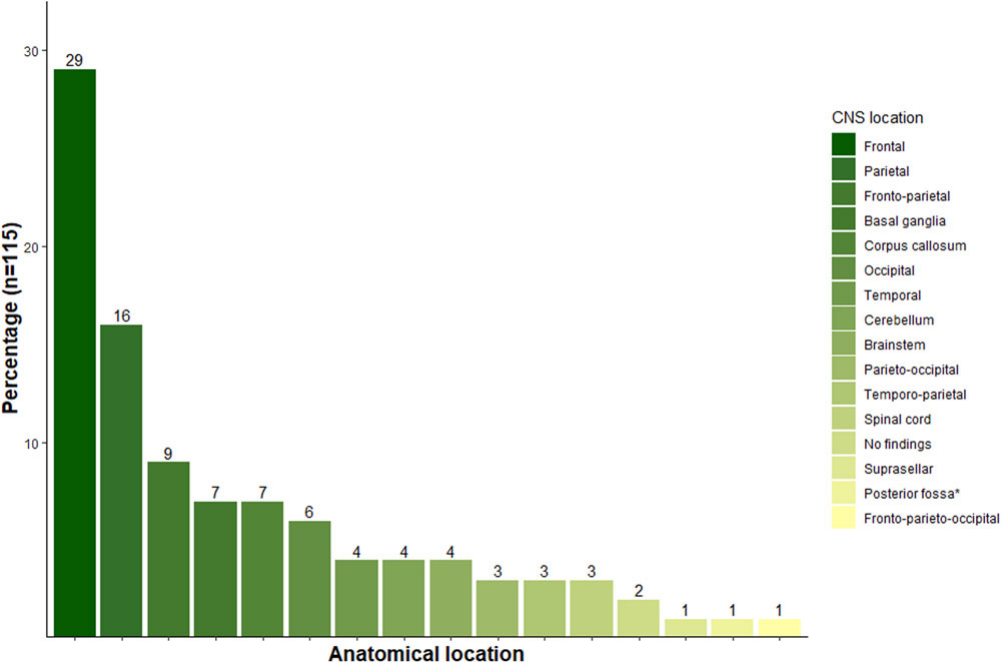
CNS distribution of lesions produced by Chagas disease. Bars represent the exact anatomical location of Chagas lesions distributed across the central nervous system (encephalon and spinal cord) in the patients retrieved by literature review. *Exact anatomical location was not mentioned.

Imaging studies were reported in 122 patients. The most common test was cerebral magnetic resonance imaging (MRI) in 41% of the patients (*n* = 50), while 34% (*n* = 41) were evaluated with a computed tomography brain scan (CT). In 24% (*n* = 29) of cases, evaluation was done with a CT scan followed by a brain MRI. A neuraxis MRI (brain and spine) or a spinal MRI was done in 2 patients, and 16 (13.1%) patients did not have a diagnostic imaging test. In 30 out of 122 (*n* = 92), there was no mention of the imaging results. Regarding to an anatomical location, 98% of patients with a CNS imaging report (90 out 92) had a documented lesion. From these patients, 89% (*n* = 80) had supratentorial lesions while only 5.5% (*n* = 5) had infratentorial lesions and 5.5% mixed cerebral lesions (supratentorial and infratentorial lesions). Furthermore, only 2 patients had a normal brain CT/MRI. In 84 patients, the exact anatomical localization of the CNS lesions was documented, with 115 lesions observed, indicating approximately 1.4 abnormal imaging findings per patient (Fig. 5). Most patients had multiple lesions which were distributed in the frontal lobe and parietal lobe, with 54% (*n* = 62) of the lesions distributed in either cerebral lobe, evidencing a higher proportion of these lesions in the frontal lobe (40%; *n* = 33). Subcortical structures including basal ganglia nuclei and commissural structures such as the corpus callosum were evenly affected, with 8 lesions documented in each area, representing 14% (*n* = 16) of the lesions. The least affected cerebral lobe was the temporal lobe, with 5.21% of lesions occurring in this area (*n* = 6). It is worth mentioning that in 1 case report, there was suprasellar involvement. It is important to highlight the prevalence of the lesions when analysing the cranial fossa compartments and the total number of lesions (*n* = 115): 86% (*n* = 99) and 9.5% (*n* = 11) were present in the anterior or middle cranial fossa and posterior cranial fossa, respectively.

### Diagnostic tests for Chagas disease in the central nervous system

Lumbar puncture was a common practice in these patients based on the suspicion of CNS pathology. Out of 138 patients, 64% (*n* = 89) underwent a lumbar puncture, while in 35% (*n* = 49) of patients, it was either not performed or not reported. Among these samples, 71% (*n* = 63) showed parasitic forms, while 29% (*n* = 26) did not. From the 89 patients who underwent lumbar puncture, 83% (*n* = 74) of the CSF samples were further analysed. CSF clinical laboratory studies were reported in 31 patients from which white blood cell count (WBC), proteins and glucose were analysed. WBCs in CSF had a median of 15 cells per mm^3^ (IQR 3–45 cells per mm^3^; reference range: 0–5; Hrishi and Sethuraman, 2019), and proteins had a median of 83 mg dL^−1^ (IQR 51–139 mg dL^−1^ [reference values: 15–40 mg dL^−1^; Hrishi and Sethuraman, 2019]). Furthermore, in 29 patients, glucose values of CSF were reported with a mean of 44.52 mg dL^−1^ (s.d. ± 21.72 mg dL^−1^; reference values: two-thirds of blood glucose; Hrishi and Sethuraman, 2019). In CSF samples, 51 out of 85 (60%) were used for parasite searching, from which 62.7% (*n* = 32) reported parasitic forms when examined (trypomastigotes). In some patients, brain biopsies or autopsies were conducted for further study. Among the population, there were 59 brain tissue biopsies which were examined. From this group, 73% (*n* = 43) were collected from surgical brain biopsy, 24% (*n* = 14) from autopsy and 3% (*n* = 2) from both procedures. Additionally, parasitic forms were found in 93% (*n* = 55) of the histopathologic samples.

### Discrete typing units reported in patients with Chagas disease in the central nervous system

Of the 138 patients reported, in only 6.52% (*n* = 9) of the cases a DTU was reported. Among the distribution of the DTUs, 4 corresponded to TcII, 2 to TcV, 2 to TcI and 1 for TcVI. Of note, one of the isolates belonging to DTU V was described as schizodeme type S3, which the literature suggests it belongs to this genotype (Carreno *et al*., 1987).

### Initial management of patients with Chagas disease in the central nervous system

An important proportion of CNS Chagas disease patients received initially empiric treatment. The 2 most important treatments were anti-toxoplasma and anti-oedema with corticosteroids. In 138 patients, treatment for toxoplasma, mostly with pyrimethamine-sulphadiazine, was initiated in 38.4% (*n* = 53) of the patients. From these 53 patients, 70% (*n* = 37) of them died. Corticosteroid treatment for brain oedema evidenced in brain imaging was used in 24% (*n* = 33) of the patients with dexamethasone being the most common drug. Among these patients, 9.4% (*n* = 13) undergone surgery from which 2 of them had a surgical decompression, and 1 had a resection of necrotic cerebral tissue. In the other 10 patients, the specific procedure was not informed.

### Mortality of patients with Chagas disease in the central nervous system

The data showed that out of 135 patients, 74% (*n* = 99) died during their hospitalization or follow-up period and 26% (*n* = 35) survived or were not reported as dead. Additionally, of the 35 patients that survived, 46% (*n* = 16) deteriorated clinically during their hospitalization.

## Discussion

CNS involvement in Chagas disease has been considered a neglected complication of this parasitic infection due to limited understanding of the disease course and its relatively low prevalence. Furthermore, it has been shown that this complication presents a remarkably high mortality rate among immunocompromised patients. (Cordova *et al*., 2008; Sztokhamer *et al*., 2010; de Almeida *et al*., 2011). Despite advancements in HIV care and the emergence of novel treatments (Cunha *et al*., 2021), migration and access to treatment has maintained HIV prevalence numbers in precarious populations (Eshraghian *et al*., 2020). The incidence of HIV in Latin America has not changed in the last decades, and AIDS-related conditions continue to be a leading cause of death in these regions (Carriquiry *et al*., 2015). Additionally, advances in cancer treatment, autoimmune disease management and organ transplants have resulted in an increased number of individuals with compromised immune systems. This, in turn, increases the risk and prevalence of patients experiencing reactivation of Chagas disease in the CNS, particularly in Chagas-endemic countries (Multani *et al*., 2019).

This systematic review provides a comprehensive analysis of 138 patients reported in the literature who have Chagas disease affecting the CNS. The study population primarily comprised middle-aged individuals, with a median age of 35 years. The majority of the patients were male and originated from Latin American countries, specifically Argentina and Brazil, where endemicity of Chagas disease is known to occur (Brenière *et al*., 2016; Pérez-Molina and Molina, 2018).

In these CNS cases, the immune status plays a significant role, with 98% of the population being immunocompromised. Most of these patients were HIV-positive who were suggested not to have treatment or poor adherence to antiretroviral therapies, as indicated by their low CD4+ T-cell counts. It is worth to mention that female patients tended to have a slightly lower CD4+ T-cell count compared to male patients, although a statistical difference was not observed. As a result of immunosuppression, most of the CNS parasite reactivation cases presented aggressively, resulting in high mortality rates. This review supports existing literature findings, indicating that patients with CD4+ T-cell counts below 200 cells per mm^3^ were associated with more severe CNS Chagas disease (Córdova *et al*., 2013; Pittella, 2013). As the immune system relies on CD4+ T helper 1 (Th1) cells for protective immunity during the acute and chronic stages of Chagas disease, it is reasonable to expect higher parasitaemia and poor disease control in immunocompromised patients (Brener and Gazzinelli, 1997; Macaluso *et al*., 2023). T-cell responses, mediated by both CD4+ and CD8+ T cells, in Chagas disease are rather complex. However, as the disease progresses, particularly in individuals with organ involvement, T-cell proliferation decreases, along with reduced polyfunctionality (such as cytokine secretion of interkeukin-2 and interferon-*γ*). This phenomenon is accompanied by an increase in immune checkpoint molecules (i.e. Programmed death ligand 1 and Cytotoxic T-lymphocyte associated protein 4), making the cellular immune response a critical variable in the disease's pathogenesis (Gómez-Olarte *et al*., 2020; Puerta *et al*., 2022).

In this review, the presence of CNS lesions was found in 98% of patients as detected through imaging. These lesions often appeared in brain MRIs as single or multiple mass-like formations, with peripheral contrast enhancement, perilesional oedema and central necrotic areas, as similar described by other authors in cases of chagasic encephalitis (Pagano *et al*., 1999; Sartori *et al*., 2007; Cordova *et al*., 2008; DiazGranados *et al*., 2009).

Although the precise mechanism of CNS invasion by the parasite remains unknown, it is hypothesized that the parasite can breach the blood–brain barrier and/or the CSF barrier in more vulnerable sites, such as leptomeningeal vessels or circumventricular organs (Kristensson, 2011). The parasite shows an apparent affinity for supratentorial structures in the anterior/middle cranial fossa, such as the frontal and parietal lobes, which is associated with inflammation leading to tissue damage. This inflammation could affect the pyramidal tract which is responsible for motor function, potentially resulting in hemiparesis or hemiplegia (Useche *et al*., 2022).

This review offers insight into some characteristics of the clinical presentation of patients infected with *T. cruzi* in the CNS, with motor deficits and seizures being the predominant manifestations. Systemic manifestations of this disease, such as fever and headaches, are non-specific and can resemble symptoms of other CNS infections and diseases. It is important to emphasize that most of the affected individuals were HIV patients. In this context, it's worth noting that they may exhibit neurological symptoms such as seizures. This can make challenging, in some cases, to conclusively attribute reported clinical manifestations solely to *T. cruzi* infection without considering the potential influence of HIV-related effects (Zaporojan *et al*., 2018).

Among the laboratory findings, a significant number of CSF samples revealed the presence of trypomastigotes, particularly in highly symptomatic patients. The literature suggests that CSF analysis may show lymphocytic pleocytosis with normal or reduced glucose levels and increased protein levels (Pérez-Molina, 2014); similar to the reported findings. However, in some cases, the CSF profile can be normal (Cordova *et al*., 2008; Pérez-Molina, 2014).

The ambiguous clinical and radiological findings are often misleading, with cerebral toxoplasmosis or CNS tumours as possible differential diagnosis. In this context, a significant proportion of patients were treated empirically with anti-toxoplasma drugs, and unfortunately, most of them did not survive (70% mortality rate). Some patients with brain oedema received treatment with glucocorticoids. However, it is important to note that the use of corticosteroids has not been proven to be effective in improving patient outcomes. Therefore, it is crucial to carefully consider the treatment options for patients with Chagas disease affecting the CNS, given the complexity of the clinical presentation and the potential for adverse effects (Pinazo *et al*., 2013). The challenging diagnosis and high mortality highlight the need for better diagnostic tools and prompt treatment of CNS Chagas disease in clinical practice.

According to the parasite genotypes, there were only 9 isolates with DTUs reported. The geographical distribution of the patients could explain the isolates reported, as 7 out of the 9 genotypes (from TcII to TcVI DTUs) are typically found in southern Latin American countries. This suggests a possible association between the geographic origin of the patients and the specific DTUs of the parasite detected during the CNS infections (Brenière *et al*., 2016). It is also worth mentioning the lack of *T. cruzi* genotyping in patients with CNS involvement, which could be further explored in future reports and studies. While there is no definitive association between DTUs and systemic manifestations of Chagas disease in humans, data from forthcoming studies could provide valuable insights and further explore this relationship.

This systematic review had several limitations: (1) published case series and case reports might not have been captured during the literature search, potentially introducing bias in evidence selection; (2) the exclusion of articles in languages other than those selected could have led to the omission of other studies; (3) the inclusion of case reports or case series with incomplete clinical variables weakened the sample size and the robustness of the analysis; and (4) a bias in the correlation between patient mortality and CNS Chagas disease may exist due to the prevalence of severe immune defects among the individuals included in the study.

## Conclusions

This review emphasizes critical clinical and paraclinical aspects that can serve as essential clues for promptly diagnosing and addressing the CNS complications of Chagas disease. For example, particular attention should be given to male middle-aged individuals coming from Chagas-endemic countries, especially those with suspected or documented immunosuppression, and clinical presentation suggesting CNS involvement. The identification of mass-like formations in brain images can further support the suspicion of CNS Chagas disease in such cases, prompting the need for a timely diagnosis. Early diagnosis is crucial for initiating appropriate treatment to avoid rapid clinical deterioration. By considering these factors and promptly addressing CNS involvement, healthcare professionals can improve patient outcomes and optimize the management of Chagas disease-related complications in the CNS.

## Supporting information

Shelton and Gonzalez supplementary material Shelton and Gonzalez supplementary material

## Data Availability

The authors confirm that the data supporting the findings of this study are available within the article and its supplementary materials.

## Supplementary material

The supplementary material for this article can be found at https://doi.org/10.1017/S0031182023001117
