# Supplementary material for: Outcomes of patients in Chagas disease of the central nervous system: a systematic review
Source: Parasitology. 2023 Nov 21;151(1):15–23. doi: 10.1017/S0031182023001117 (PMC10941035; doi:10.1017/S0031182023001117)
Supplement: Shelton and Gonzalez supplementary material [file S0031182023001117sup001.docx]

**Supplementary material 1. Datasheet of extracted data from selected articles**

| Reference | Number of cases | Age (years) | Gender | Country | Immunosuppression | HIV | CD4 Count | Images | Findings | CNS Location | CNS clinical signs | Systemic signs/symptoms | Brain biopsy pathology | CSF | Microscopic examination | Cytochemical | DTU's | Surgical treatment | Toxoplasma treatment | Corticoid treatment | Clinical deterioration | Mortality |
| --- | --- | --- | --- | --- | --- | --- | --- | --- | --- | --- | --- | --- | --- | --- | --- | --- | --- | --- | --- | --- | --- | --- |
| (Reimer-McAtee *et al.*, 2021) | 4 |  |  |  |  |  |  |  |  |  |  |  |  |  |  |  |  |  |  |  |  |  |
| 1 | | 22 | Masculine | Bolivia | Yes | Yes | 52 cells/mm^3^ | CT | Infratentorial | Right posterior fossa | Yes | Yes | NR | NR | NR | NR | NR | NR | NR | NR | NR | NR |
| 2 | | 27 | Masculine | Bolivia | Yes | Yes | 15 cells/mm^3^ | NR | NR | NR | NR | Yes | NR | NR | NR | NR | NR | NR | NR | NR | NR | NR |
| 3 | | 26 | Masculine | Bolivia | Yes | Yes | 29 cells/mm^3^ | NR | NR | NR | NR | Yes | NR | NR | NR | NR | NR | NR | NR | NR | Yes | Yes |
| 4 | | 26 | Masculine | Bolivia | Yes | Yes | 3 cells/mm^3^ | NR | NR | NR | NR | Yes | NR | NR | NR | NR | NR | NR | NR | NR | NR | NR |
| (Lopez-Albizu *et al.*, 2021) | 1 | 46 | Masculine | Argentina | Yes | Yes | NR | MRI | Supratentorial | Right parieto-occipital-subcortical lesion | Yes | NR | Biopsy, parasitic forms | NR | NR | NR | NR | NR | NR | NR | NR | NR |
| (Fernández *et al.*, 2021) | 6 |  |  |  |  |  |  |  |  |  |  |  |  |  |  |  |  |  |  |  |  |  |
| 1 | | 39 | Feminine | Argentina | Yes | Yes | 9 cells/mm^3^ | NR | NR | NR | Yes | NR | NR | Yes | NR parasites | NR | NR | NR | Yes | Yes | NR | NR |
| 2 | | 65 | Masculine | Argentina | Yes | Yes | 10 cells/mm^3^ | NR | NR | NR | Yes | NR | NR | Yes | Parasitic forms | NR | NR | NR | Yes | Yes | Yes | Yes |
| Reference | Number of cases | Age (years) | Gender | Country | Immunosuppression | HIV | CD4 Count | Images | Findings | CNS Location | CNS clinical signs | Systemic signs/symptoms | Brain biopsy pathology | CSF | Microscopic examination | Cytochemical | DTU's | Surgical treatment | Toxoplasma treatment | Corticoid treatment | Clinical deterioration | Mortality |
| 3 | | 42 | Masculine | Argentina | Yes | Yes | 7 cells/mm^3^ | NR | NR | NR | Yes | NR | NR | Yes | Parasitic forms | NR | NR | NR | Yes | Yes | Yes | Yes |
| 4 | | 69 | Masculine | Argentina | Yes | Yes | 19 cells/mm^3^ | NR | NR | NR | Yes | NR | NR | Yes | Parasitic forms | NR | NR | NR | Yes | Yes | Yes | Yes |
| 5 | | 65 | Feminine | Argentina | Yes | Yes | 16 cells/mm^3^ | NR | NR | NR | Yes | NR | NR | Yes | Parasitic forms | NR | NR | NR | Yes | NR | NR | NR |
| (Koff *et al.*, 2020) | 1 | 64 | Masculine | USA | Yes (transplant) | NR | NR | MRI | NR | NR | Yes | Yes | NR | Yes | Parasitic forms | Yes | NR | NR | Yes | Yes | Yes | Yes |
| (Guidetto *et al.*, 2019) | 2 |  |  |  |  |  |  |  |  |  |  |  |  |  |  |  |  |  |  |  |  |  |
| 1 | | 53 | Feminine | Argentina | Yes | Yes | 5 cells/mm^3^ | CT | Supratentorial | Temporo-parietal | NR | Yes | NR | Yes | Parasitic forms | Yes | NR | NR | NR | NR | NR | NR |
| 2 | | 38 | Feminine | Argentina | Yes | Yes | 53 cells/mm^3^ | MRI | Supratentorial | Frontal | Yes | NR | NR | Yes | NR parasites | Yes | NR | NR | NR | NR | NR | NR |
| (Montero *et al.*, 2019) | 1 | 62 | Masculine | Bolivia | Yes (transplant) | NR | NA | MRI | Supratentorial | Corpus callosum | Yes | Yes | Biopsy, parasitic forms | Yes | NR parasites | NR | NR | NR | Yes | Yes | Yes | NR |
| (Multani *et al.*, 2019) | 1 | 31 | Masculine | El Salvador | Yes | Yes | 60 cells/mm^3^ | MRI | Supratentorial | Corpus callosum and parietal-occipital subcortical white matter | Yes | Yes | Biopsy, parasitic forms | NR | NR | NR | NR | NR | Yes | NR | Yes | NR |
| (Kaushal *et al.*, 2019) | 1 | 88 | Feminine | USA | Yes (autoimmune disease treatment) | NR | 34 cells/mm^3^ | MRI | Supratentorial | Frontoparietal region | Yes | NR | Biopsy, parasitic forms | Yes | NR parasites | Yes | NR | NR | NR | NR | Yes | Yes |
| (Fernandes *et al.*, 2017) | 1 | 57 | Masculine | Brazil | NR | NR | 480 cells/mm^3^ | CT | Supratentorial | Frontoparietal region | Yes | NR | NR | Yes | Parasitic forms | Yes | NR | NR | NR | NR | Yes | Yes |
| Reference | Number of cases | Age (years) | Gender | Country | Immunosuppression | HIV | CD4 Count | Images | Findings | CNS Location | CNS clinical signs | Systemic signs/symptoms | Brain biopsy pathology | CSF | Microscopic examination | Cytochemical | DTU's | Surgical treatment | Toxoplasma treatment | Corticoid treatment | Clinical deterioration | Mortality |
| (Alyemni *et al.*, 2017) | 1 | 47 | Masculine | Mexico | Yes | Yes | 18 cells/mm^3^ | MRI | Supratentorial and infratentorial | Frontal and midbrain-pons junction | Yes | NR | Biopsy, parasitic forms | Yes | NR parasites | Yes | NR | NR | Yes | NR | Yes | Yes |
| (Camargos *et al.*, 2017) | 1 | 39 | Feminine | Brazil | Yes (transplant) | NR | NR | MRI | Supratentorial | Frontoparietal | Yes | Yes | Biopsy, parasitic forms | NR | NR | NR | NR | NR | NR | NR | Yes | Yes |
| (Simioli *et al.*, 2017) | 1 | 36 | Feminine | Argentina | Yes | Yes | 79 cells/mm^3^ | MRI | Supratentorial and infratentorial | Frontal and cerebellum with extension to cerebral trunk | Yes | NR | NR | Yes | NR parasites | Yes | NR | NR | Yes | NR | Yes | Yes |
| (Fica Cubillos *et al.*, 2017) | 1 | 60 | Masculine | Chile | Yes | Yes | 74 cells/mm^3^ | CT and MRI | Supratentorial and infratentorial | Frontal, frontoparietal and cerebellar | Yes | Yes | NR | Yes | NR parasites | Yes | NR | NR | Yes | Yes | Yes | Yes |
| (Chalub *et al.*, 2017) | 1 | 32 | Feminine | Argentina | Yes | Yes | NR | MRI | Supratentorial | Frontoparietal, occipital and corpus callosum | Yes | Yes | Biopsy, parasitic forms | NR | NR | NR | NR | NR | Yes | Yes | Yes | Yes |
| (Buccheri *et al.*, 2015) | 1 | 42 | Feminine | Brazil | Yes | Yes | 318 cells/mm^3^ | CT and MRI | Supratentorial | Temporo-parietal | Yes | Yes | NR | Yes | Parasitic forms | Yes | TcII | NR | Yes | Yes | Yes | NR |
| (Spadafora *et al.*, 2014) | 1 | 38 | Masculine | Venezuela | Yes | Yes | 200 cells/mm^3^ | CT | Supratentorial | Frontal lobe and basal nuclei | Yes | Yes | Autopsy, parasitic forms | Yes | Parasitic forms | NR | NR | NR | Yes | NR | Yes | Yes |
| (Hernández *et al.*, 2014) | 1 | 34 | Feminine | Colombia | Yes | Yes | NR | CT and MRI | Supratentorial | Frontal | NR | Yes | Autopsy, parasitic forms | Yes | Parasitic forms | Yes | TcI | NR | Yes | NR | Yes | Yes |
| (Cicora *et al.*, 2014) | 1 | 27 | Masculine | Argentina | Yes (transplant) | NR | NR | CT | Supratentorial | Frontal | NR | Yes | NR | Yes | NR parasites | Yes | NR | NR | NR | NR | Yes | Yes |
| (Castillo *et al.*, 2014) | 1 | NR | Masculine | Argentina | Yes | Yes | 100 cells/mm^3^ | MRI | Supratentorial | Corpus callosum | NR | NR | NR | Yes | Parasitic forms | Yes | NR | NR | NR | NR | NR | NR |
| (Yasukawa *et al.*, 2014) | 1 | 49 | Feminine | Honduras | Yes | Yes | 38 cells/mm^3^ | MRI | Supratentorial | Frontal and parietal | Yes | Yes | Biopsy, parasitic forms | Yes | Parasitic forms | Yes | NR | NR | Yes | Yes | Yes | NR |
| (Bisio *et al.*, 2013) | 1 | 33 | Feminine | Argentina | Yes | Yes | 18 cells/mm^3^ | MRI | Supratentorial | NR | Yes | Yes | NR | NR | NR | NR | TcV | NR | NR | NR | NR | NR |
| Reference | Number of cases | Age (years) | Gender | Country | Immunosuppression | HIV | CD4 Count | Images | Findings | CNS Location | CNS clinical signs | Systemic signs/symptoms | Brain biopsy pathology | CSF | Microscopic examination | Cytochemical | DTU's | Surgical treatment | Toxoplasma treatment | Corticoid treatment | Clinical deterioration | Mortality |
| (Castrillon, 2013) | 1 | 42 | Masculine | Argentina | Yes | Yes | 100 cells/mm^3^ | CT | Supratentorial | Frontal | Yes | Yes | Biopsy, parasitic forms | NR | NR | NR | NR | NR | NR | NR | NR | NR |
| (Bestetti *et al.*, 2013) | 1 | 39 | Feminine | Brazil | Yes (transplant) | NR | NR | MRI | Supratentorial | Frontal and basal nuclei | Yes | NR | Biopsy, parasitic forms | Yes | NR parasites | Yes | NR | NR | NR | NR | NR | NR |
| (Bernabeu, Alcaraz-Mateos and Lopez-Celada, 2013) | 1 | 48 | Masculine | Argentina | Yes | Yes | NR | MRI | Supratentorial | Frontal and occipital | NR | Yes | Biopsy, parasitic forms | NR | NR | NR | NR | Yes | Yes | NR | NR | NR |
| (Menghi, Gatta and Arcavi, 2010) | 1 | 35 | Masculine | Argentina | Yes | Yes | 18 cells/mm^3^ | MRI | Supratentorial | Frontoparietal | Yes | Yes | NR | Yes | Parasitic forms | NR | NR | NR | NR | NR | NR | NR |
| (Olga, 2010) | 1 | 36 | Masculine | Chile | Yes | Yes | NR | CT | Supratentorial | Frontoparietal | Yes | Yes | NR | Yes | Parasitic forms | Yes | NR | NR | NR | NR | NR | NR |
| (Cohen *et al.*, 2010) | 1 | 15 | Feminine | Argentina | Yes (chemotherapy) | NR | NR | CT and MRI | Supratentorial | Occipital | NR | Yes | NR | Yes | NR parasites | NR | NR | NR | NR | NR | NR | NR |
| (Campo *et al.*, 2010) | 1 | 38 | Feminine | Honduras | Yes | Yes | 104 cells/mm^3^ | MRI | Supratentorial | Parietal | Yes | Yes | Biopsy, parasitic forms | NR | NR | NR | NR | NR | Yes | NR | Yes | Yes |
| (Sztokhamer *et al.*, 2010) | 24 | Mean 31 years (22-52) | 22 Masculine, 2 Feminine | Argentina | Yes | Yes | (#9) <100 cells/mm^3^ (#2) 100-200 cells/mm^3^ (#1) 270 cells/mm^3^ | MRI | NR | NR | Yes 4/16 | Yes 13/16 | Autopsy 6/24, parasitic forms (6/6) | Yes 14/24 | Parasitic forms 10/14 | NR | NR | NR | NR | NR | Yes 21/24 | Yes 21/24 |
| (Verdú *et al.*, 2009) | 1 | 35 | Masculine | Argentina | Yes | Yes | NR | CT and MRI | Supratentorial | NR | NR | Yes | Biopsy, parasitic forms | NR | NR | NR | NR | NR | NR | NR | Yes | NR |
| (DiazGranados *et al.*, 2009) | 1 | 26 | Feminine | Colombia | Yes | Yes | 189 cells/mm^3^ | CT | Supratentorial | Basal ganglia | Yes | Yes | Biopsy, NR | Yes | Parasitic forms | Yes | NR | NR | Yes | NR | Yes | NR |
| (Moretta *et al.*, 2009) | 1 | 35 | Masculine | Argentina | Yes | Yes | 18 cells/mm^3^ | CT and MRI | Supratentorial | Temporo-parietal | Yes | Yes | NR | Yes | Parasitic forms | Yes | NR | NR | Yes | NR | Yes | NR |
| Reference | Number of cases | Age (years) | Gender | Country | Immunosuppression | HIV | CD4 Count | Images | Findings | CNS Location | CNS clinical signs | Systemic signs/symptoms | Brain biopsy pathology | CSF | Microscopic examination | Cytochemical | DTU's | Surgical treatment | Toxoplasma treatment | Corticoid treatment | Clinical deterioration | Mortality |
| (De Almeida *et al.*, 2009) | 1 | 33 | Masculine | Brazil | Yes | Yes | 9 cells/mm^3^ | MRI | Supratentorial | Frontal, parietal, occipital | Yes | Yes | NR | Yes | Parasitic forms | NR | NR | NR | NR | NR | Yes | NR |
| (Sica, Gargiullo and Papayanis, 2008) | 3 |  |  |  |  |  |  |  |  |  |  |  |  |  |  |  |  |  |  |  |  |  |
| 1 | | 28 | Masculine | Brazil | Yes | Yes | NR | MRI | Supratentorial | Parietal | Yes | Yes | Biopsy, parasitic forms | NR | NR | NR | NR | NR | Yes | NR | Yes | Yes |
| 2 | | 51 | Masculine | Brazil | Yes | Yes | 46 cells/mm^3^ | MRI | Supratentorial | NR | Yes | NR | Biopsy, parasitic forms | Yes | Parasitic forms | NR | NR | NR | Yes | NR | Yes | Yes |
| 3 | | 35 | Masculine | Brazil | Yes | Yes | 551 cells/mm^3^ | CT and MRI | Supratentorial | Parietal | Yes | Yes | Biopsy, parasitic forms | NR | NR | NR | NR | NR | Yes | NR | Yes | NR |
| (Cordova *et al.*, 2008) | 15 | Median 33 years (25-54) | 14 Masculine, 1 Feminine | Argentina | Yes | Yes | Median 64 cells/mm^3^ (1-240) | Yes (14/15); 5 CT, 1 MRI, 8 CT+MRI | Single supratentorial 7/12  Multiple supratentorial 3/12  Multiple infratentorial 2/12 Normal imaging 2/12 | Brain s 10/12 (3 frontal), basal ganglia/thalamus 2/12, Brainstem 1/12  Cerebellum 2/2 | Yes 15/15 | 1. Headache 11/15 2. Fever 9/15 | Biopsy 3/15, parasitic forms (3/3) | Yes (13/15) NR CI (2/15) | Parasitic forms (11/13) | Yes | NR | NR | Yes 1/15 NR 14/15 | Yes 3/15 | Yes 1/15 | Yes 11/14  NR 3/14 |
| (Burgos *et al.*, 2008) | 1 | 41 | Masculine | Bolivia | Yes | Yes | 61 cells/mm^3^ | MRI vertebral column | Infratentorial | Spinal cord | Yes | Yes | NR | Yes | NR parasites | NR | TcI | NR | NR | NR | Yes | Yes |
| (Fernandez de Castro, Sanchez and Cortes, 2008) | 1 | 40 | Masculine | Colombia | Yes (transplant) | NR | NR | CT and MRI | Supratentorial | Frontal | NR | Yes | Biopsy, parasitic forms | Yes | NR parasites | Yes | NR | NR | NR | NR | NR | NR |
| (De Medeiros, Guerra and De Lacerda, 2008) | 1 | 16 | Feminine | Brazil | NR | NR | NR | CT | Supratentorial | Frontal, parietal, and temporal | Yes | Yes | NR | Yes | NR parasites | Yes | NR | NR | NR | Yes | Yes | NR |
| (Marchiori *et al.*, 2007) | 1 | 46 | Masculine | Brazil | Yes (transplant) | NR | NR | CT and MRI | Supratentorial | Frontoparietal | Yes | Yes | Biopsy, parasitic forms | Yes | Parasitic forms | Yes | NR | Yes (resection of necrotic cerebral tissue) | NR | NR | Yes | Yes |
| (Nijjar and Del Bigio, 2007) | 1 | 40 | Masculine | Canada | Yes | NR | 4 cells/mm^3^ | CT | Supratentorial | Frontal | Yes | Yes | Biopsy, parasitic forms | NR | NR | NR | NR | Yes (surgical decompression) | NR | NR | Yes | Yes |
| Reference | Number of cases | Age (years) | Gender | Country | Immunosuppression | HIV | CD4 Count | Images | Findings | CNS Location | CNS clinical signs | Systemic signs/symptoms | Brain biopsy pathology | CSF | Microscopic examination | Cytochemical | DTU's | Surgical treatment | Toxoplasma treatment | Corticoid treatment | Clinical deterioration | Mortality |
| (Sartori *et al.*, 2007) | 4 |  |  |  |  |  |  |  |  |  |  |  |  |  |  | Yes |  |  |  |  |  |  |
| 1 | | 47 | Masculine | Brazil | Yes | Yes | NR | NR | NR | NR | Yes | Yes | Autopsy, parasitic forms | Yes | NR parasites | NR | NR | NR | NR | NR | Yes | Yes |
| 2 | | 41 | Feminine | Brazil | Yes | Yes | 37 cells/mm^3^ | NR | NR | NR | Yes | Yes | NR | Yes | Parasitic forms | NR | NR | NR | NR | NR | Yes | Yes |
| 3 | | 29 | Masculine | Brazil | Yes | Yes | NR | NR | NR | NR | Yes | Yes | NR | Yes | Parasitic forms | NR | NR | NR | NR | NR | Yes | Yes |
| 4 | | 33 | Masculine | Brazil | Yes | Yes | 26 cells/mm^3^ | NR | NR | NR | Yes | Yes | NR | Yes | Parasitic forms | NR | NR | NR | NR | NR | Yes | Yes |
| (Lambert *et al.*, 2006) | 1 | 56 | Masculine | Mexico | Yes | Yes | 37 cells/mm^3^ | MRI | Supratentorial and infratentorial | Frontoparietal, temporal and conus medullaris | Yes | Yes | Biopsy, parasitic forms | NR | NR | NR | NR | NR | Yes | NR | Yes | Yes |
| (Cor José Antonio Frías Salcedo *et al.*, 2006) | 1 | 58 | Feminine | Mexico | Yes | Yes | NR | CT | Supratentorial | Parietal | Yes | Yes | NR | Yes | Parasitic forms | Yes | NR | NR | NR | NR | Yes | Yes |
| (Lury and Castillo, 2005) | 1 | 56 | Masculine | USA | Yes | Yes | 50 cells/mm^3^ | MRI of brain and spine | Supratentorial and infratentorial | Corpus callosum, cerebellum, subcortical regions, spinal cord | Yes | Yes | Biopsy, parasitic forms | NR | NR | NR | NR | NR | Yes | NR | Yes | Yes |
| (Valerga *et al.*, 2005) | 1 | 33 | Masculine | Argentina | Yes | Yes | 106 cells/mm^3^ | MRI | Supratentorial | Parietal, occipital | Yes | Yes | Biopsy and autopsy, parasitic forms | NR | NR | NR | NR | NR | Yes | NR | Yes | Yes |
| (Burgos *et al.*, 2005) | 1 | 29 | Masculine | Argentina | Yes | Yes | NR | MRI | Supratentorial | Frontal, parietal | Yes | Yes | Biopsy, NR | Yes | NR parasites | NR | TcII | NR | Yes | NR | NR | NR |
| (Yoo *et al.*, 2004) | 1 | 22 | Masculine | El Salvador | Yes | Yes | 77 cells/mm^3^ | MRI | Supratentorial | Internal capsule | Yes | Yes | NR | Yes | Parasitic forms | Yes | NR | NR | Yes | Yes | Yes | Yes |
| (Madalosso *et al.*, 2004) | 1 | 52 | Masculine | Brazil | Yes | Yes | NR | NR | NR | NR | Yes | Yes | Autopsy, NR | Yes | Parasitic forms | Yes | NR | NR | NR | NR | Yes | Yes |
| Reference | Number of cases | Age (years) | Gender | Country | Immunosuppression | HIV | CD4 Count | Images | Findings | CNS Location | CNS clinical signs | Systemic signs/symptoms | Brain biopsy pathology | CSF | Microscopic examination | Cytochemical | DTU's | Surgical treatment | Toxoplasma treatment | Corticoid treatment | Clinical deterioration | Mortality |
| (Choi *et al.*, 2004) | 1 | 28 | Masculine | Mexico | NR | NR | 621 cells/mm^3^ | CT and MRI | Supratentorial | Suprasellar (hypothalamus, thalamus) | NR | Yes | Biopsy and autopsy, parasitic forms | Yes | NR parasites | Yes | NR | NR | NR | Yes | Yes | Yes |
| (Marques de Brito, Pires and Pacheco, 2003) | 1 | NR | NR | Brazil | Yes | Yes | 102 cells/mm^3^ | NR | NR | NR | NR | NR | NR | NR | NR | NR | TcII | NR | NR | NR | NR | Yes |
| (Lages-Silva *et al.*, 2002) | 1 | 63 | Feminine | Brazil | Yes | Yes | 67 cells/mm^3^ | CT | NR | NR | Yes | Yes | NR | Yes | Parasitic forms | NR | TcII | NR | Yes | NR | Yes | NR |
| (de Oliveira Santos *et al.*, 2002) | 1 | 26 | Masculine | Brazil | Yes | Yes | NR | NR | NR | NR | Yes | NR | NR | Yes | Parasitic forms | Yes | NR | NR | NR | NR | Yes | Yes |
| (Antunes *et al.*, 2002) | 1 | 36 | Feminine | Brazil | Yes | Yes | 8 cells/mm^3^ | CT | Supratentorial | Corpus callosum | Yes | Yes | NR | Yes | Parasitic forms | Yes | NR | NR | NR | NR | Yes | Yes |
| (Corti *et al.*, 2000) | 1 | 25 | Masculine | Argentina | Yes | Yes | 1 cell/mm^3^ | CT | Supratentorial | Frontoparietal | Yes | Yes | Autopsy, parasitic forms | NR | NR | NR | NR | NR | Yes | Yes | Yes | Yes |
| (Pagano *et al.*, 1999) | 10 |  | 9 masculine, 1 feminine |  |  |  | 126-63 cells/mm^3^ (8/10) | Yes (10/10); 1 CT, 1 MRI, 8 CT + MRI |  |  |  |  | Biopsy 8/10, parasitic forms (8/8) | Yes (3/10) | Parasitic forms 2/3 |  |  | Yes 6/10 (neurosurgical decompression) | Yes 9/10 | Yes 10/10 | Yes 10/10 | Yes 9/10 |
| 1 | | 32 | NR | Argentina | Yes | Yes | NR | NR | Supratentorial | Right parietal | Yes | Yes | NR | NR | NR | NR | NR | NR | NR | NR | NR | NR |
| 2 | | 33 | NR | Argentina | Yes | Yes | NR | NR | Supratentorial | Left frontal | Yes | Yes | NR | NR | NR | NR | NR | NR | NR | NR | NR | NR |
| 3 | | 21 | NR | Argentina | Yes | Yes | NR | NR | Supratentorial | Right frontal + Left parietal | Yes | NR | NR | NR | NR | NR | NR | NR | NR | NR | NR | NR |
| 4 | | 25 | NR | Argentina | Yes | Yes | NR | NR | Supratentorial | Right temporal | Yes | Yes | NR | NR | NR | NR | NR | NR | NR | NR | NR | NR |
| 5 | | 28 | NR | Argentina | Yes | Yes | NR | NR | Supratentorial | Left frontal | Yes | NR | NR | NR | NR | NR | NR | NR | NR | NR | NR | NR |
| Reference | Number of cases | Age (years) | Gender | Country | Immunosuppression | HIV | CD4 Count | Images | Findings | CNS Location | CNS clinical signs | Systemic signs/symptoms | Brain biopsy pathology | CSF | Microscopic examination | Cytochemical | DTU's | Surgical treatment | Toxoplasma treatment | Corticoid treatment | Clinical deterioration | Mortality |
| 6 | | 25 | NR | Argentina | Yes | Yes | NR | NR | Supratentorial | Left frontal | Yes | NR | NR | NR | NR | NR | NR | NR | NR | NR | NR | NR |
| 7 | | 50 | NR | Argentina | Yes | Yes | NR | NR | Supratentorial | Left frontal | Yes | NR | NR | NR | NR | NR | NR | NR | NR | NR | NR | NR |
| 8 | | 42 | NR | Argentina | Yes | Yes | NR | NR | Supratentorial | Right frontal-parieto-occipital | Yes | NR | NR | NR | NR | NR | NR | NR | NR | NR | NR | NR |
| 9 | | 37 | NR | Argentina | Yes | Yes | NR | NR | Supratentorial | Right parieto-occipital | Yes | Yes | NR | NR | NR | NR | NR | NR | NR | NR | NR | NR |
| 10 | | 29 | NR | Argentina | Yes | Yes | NR | NR | Supratentorial | Right basal ganglia + left mesencephalic + left pontine | Yes | Yes | NR | NR | NR | NR | NR | NR | NR | NR | NR | NR |
| (Silva *et al.*, 1999) | 5 |  |  |  |  |  |  |  |  |  |  |  |  |  |  |  |  |  |  |  |  |  |
| 1 | | 36 | Masculine | Brazil | Yes | Yes | 23 cells/mm^3^ | CT | NR | NR | Yes | NR | NR | Yes | Parasitic forms | Yes | NR | NR | Yes | NR | Yes | Yes |
| 2 | | 29 | Masculine | Brazil | Yes | Yes | 46 cells/mm^3^ | CT | Supratentorial | Corpus callosum | Yes | NR | NR | Yes | Parasitic forms | Yes | NR | NR | Yes | NR | Yes | Yes |
| 3 | | 25 | Masculine | Brazil | Yes | Yes | NR | CT | Supratentorial | Corpus callosum | Yes | Yes | NR | Yes | Parasitic forms | Yes | NR | NR | Yes | NR | Yes | Yes |
| 4 | | 24 | Feminine | Brazil | Yes | Yes | NR | CT | NR | NR | Yes | Yes | NR | Yes | Parasitic forms | Yes | NR | NR | Yes | NR | Yes | Yes |
| 5 | | 25 | Masculine | Brazil | Yes | Yes | 185 cells/mm^3^ | CT | Supratentorial | Basal ganglia | Yes | NR | NR | Yes | Parasitic forms | Yes | NR | NR | Yes | NR | Yes | Yes |
| (De Sousa Dos Santos *et al.*, 1999) | 1 | 47 | Masculine | Brazil | Yes | Yes | NR | CT and MRI | Supratentorial | Occipital, temporal, parietal, periventricular | NR | Yes | Autopsy, parasitic forms | Yes | NR parasites | Yes | NR | NR | NR | NR | Yes | Yes |
| Reference | Number of cases | Age (years) | Gender | Country | Immunosuppression | HIV | CD4 Count | Images | Findings | CNS Location | CNS clinical signs | Systemic signs/symptoms | Brain biopsy pathology | CSF | Microscopic examination | Cytochemical | DTU's | Surgical treatment | Toxoplasma treatment | Corticoid treatment | Clinical deterioration | Mortality |
| (Cohen *et al.*, 1998) | 1 | 30 | Masculine | Argentina | Yes | Yes | NR | CT | Supratentorial | Parietal | Yes | Yes | Biopsy, parasitic forms | NR | NR | NR | NR | NR | Yes | Yes | Yes | Yes |
| (Montero *et al.*, 1998) | 2 |  |  |  |  |  |  |  |  |  |  |  |  |  |  |  |  |  |  |  |  |  |
| 1 | | 38 | NR | Argentina | Yes | Yes | 125 cells/mm^3^ | CT | Supratentorial | Parietal | Yes | NR | Biopsy, parasitic forms | Yes | Parasitic forms | NR | NR | NR | Yes | NR | Yes | Yes |
| 2 | | 29 | NR | Argentina | Yes | Yes | 62 cells/mm^3^ | CT | Supratentorial | Frontal | Yes | Yes | NR | Yes | Parasitic forms | NR | NR | NR | Yes | NR | Yes | Yes |
| (Pacheco *et al.*, 1998) | 1 | 27 | Masculine | Brazil | Yes | Yes | 200 cells/mm^3^ | CT | NR | NR | NR | Yes | NR | NR | NR | NR | TcVI | NR | NR | NR | Yes | Yes |
| (Ferreira *et al.*, 1997) | 1 | 27 | Masculine | Brazil | Yes | Yes | 102 cells/mm^3^ | CT | NR | NR | NR | Yes | NR | NR | NR | NR | NR | NR | NR | NR | Yes | Yes |
| (Salgado *et al.*, 1996) | 1 | 73 | Masculine | Brazil | Yes (leukaemia) | NR | NR | CT | Supratentorial | Occipital and parietal | Yes | Yes | NR | Yes | Parasitic forms | Yes | NR | NR | NR | Yes | Yes | NR |
| (Pimentel, Handfas and Carmignani, 1996) | 1 | 47 | Masculine | Brazil | Yes | Yes | NR | CT | Supratentorial | Frontal and parietal | Yes | Yes | NR | Yes | Parasitic forms | Yes | NR | NR | NR | Yes | Yes | Yes |
| (Di Lorenzo *et al.*, 1996) | 3 |  |  |  |  |  |  |  |  |  |  |  |  |  |  |  |  |  |  |  |  |  |
| 1 | | 32 | NR | Argentina | Yes | Yes | NR | CT and MRI | Supratentorial | NR | Yes | NR | Biopsy, parasitic forms | NR | NR | NR | NR | Yes | Yes | NR | Yes | NR |
| 2 | | 32 | NR | Argentina | Yes | Yes | NR | CT and MRI | Supratentorial | Frontal lobe | Yes | Yes | Biopsy, parasitic forms | NR | NR | NR | NR | Yes | Yes | NR | Yes | NR |
| 3 | | 9 | Masculine | Argentina | Yes (leukaemia) | NR | N/R | CT | Supratentorial | Frontal lobe | Yes | NR | Biopsy, parasitic forms | NR | NR | NR | NR | Yes | NR | Yes | Yes | Yes |
| Reference | Number of cases | Age (years) | Gender | Country | Immunosuppression | HIV | CD4 Count | Images | Findings | CNS Location | CNS clinical signs | Systemic signs/symptoms | Brain biopsy pathology | CSF | Microscopic examination | Cytochemical | DTU's | Surgical treatment | Toxoplasma treatment | Corticoid treatment | Clinical deterioration | Mortality |
| (Solari *et al.*, 1993) | 1 | 31 | Masculine | Chile | Yes | Yes | 35 cells/mm^3^ | CT | Supratentorial | Parietal and frontal | Yes | Yes | Biopsy, parasitic forms | NR | NR | NR | TcV | NR | Yes | NR | Yes | NR |
| (Nishioka *et al.*, 1993) | 1 | 33 | Masculine | Brazil | Yes | Yes | 382 cells/mm^3^ | CT | Supratentorial | Temporo-occipital | NR | Yes | Autopsy, NR | Yes | Parasitic forms | Yes | NR | NR | NR | Yes | Yes | Yes |
| (Rocha *et al.*, 1993) | 1 | 52 | Masculine | Brazil | Yes | Yes | NR | CT | Supratentorial | Frontal and parietal | Yes | Yes | Autopsy, parasitic forms | Yes | NR parasites | Yes | NR | NR | NR | NR | Yes | Yes |
| (Cardozo *et al.*, 1992) | 1 | 33 | Masculine | Uruguay | Yes | Yes | NR | CT | Supratentorial | Frontal | Yes | Yes | NR | Yes | Parasitic forms | Yes | NR | NR | NR | NR | Yes | Yes |
| (Gluckstein, Ciferri and Ruskin, 1992) | 1 | 32 | Masculine | El Salvador | Yes | Yes | 45 cells/mm^3^ | CT | Supratentorial | Temporal | Yes | Yes | Biopsy, parasitic forms | NR | NR | NR | NR | NR | Yes | Yes | Yes | Yes |
| (Gallo *et al.*, 1992) | 1 | 26 | Feminine | Brazil | Yes | Yes | NR | CT | Infratentorial | Midbrain | Yes | Yes | NR | Yes | Parasitic forms | Yes | NR | NR | NR | NR | Yes | Yes |
| (Del Castillo *et al.*, 1990) | 1 | 19 | Masculine | Argentina | Yes | Yes | NR | CT | Supratentorial | Frontal | NR | Yes | Biopsy, parasitic forms | Yes | NR parasites | NR | NR | Yes | NR | NR | Yes | Yes |
| (Oddó *et al.*, 1992) | 1 | 31 | Masculine | Chile | Yes | Yes | NR | CT | Supratentorial | Frontal and parietal | Yes | Yes | Biopsy, parasitic forms | NR | NR | NR | NR | NR | Yes | NR | Yes | NR |

^NR, not reported; CT, brain computed tomography scan; MRI, brain magnetic resonance imaging.^

# **References**

**De Almeida, E. A. *et al.*** (2009) ‘Aetiological treatment with itraconazole or ketoconazole in individuals with Trypanosoma cruzi/HIV co-infection’, *Annals of tropical medicine and parasitology*. Ann Trop Med Parasitol, 103(6), pp. 471–476. doi: 10.1179/000349809X12459740922174.

**Alyemni, D. *et al.*** (2017) ‘Histopathologic identification of Trypanosoma cruzi (Chagas’) encephalitis in an AIDS patient’, *Human Pathology: Case Reports*. Elsevier, 7, pp. 23–26. doi: 10.1016/J.EHPC.2016.06.002.

**Antunes, A. C. M. *et al.*** (2002) ‘Cerebral trypanosomiasis and AIDS’, *Arquivos de Neuro-Psiquiatria*. Academia Brasileira de Neurologia - ABNEURO, 60(3 B), pp. 730–733. doi: 10.1590/S0004-282X2002000500009.

**Bernabeu, A., Alcaraz-Mateos, E. and Lopez-Celada, S.** (2013) ‘Usefulness of Magnetic Resonance Spectroscopy in the Assessment of Brain Chagas Disease: A Case Report’, *Journal of Neurology Research*, 3(5), pp. 150–154. doi: 10.4021/JNR.V3I5.237.

**Bestetti, R. B. *et al.*** (2013) ‘Trypanosoma cruzi infection reactivation manifested by encephalitis in a Chagas heart transplant recipient’, *International Journal of Cardiology*. Elsevier Ireland Ltd, 163(1), pp. e7–e8. doi: 10.1016/j.ijcard.2012.06.102.

**Bisio, M. *et al.*** (2013) ‘Benznidazole Treatment of Chagasic Encephalitis in Pregnant Woman with AIDS’, *Emerging Infectious Diseases*. Centers for Disease Control and Prevention, 19(9), p. 1490. doi: 10.3201/EID1909.130667.

**Buccheri, R. *et al.*** (2015) ‘Chagasic meningoencephalitis in an HIV infected patient with moderate immunosuppression: Prolonged survival and challenges in the HAART era.’, *Revista do Instituto de Medicina Tropical de São Paulo*. Instituto De Medicina Tropical De Sao Paulo, 57(6), p. 531. doi: 10.1590/S0036-46652015000600014.

**Burgos, J. M. *et al.*** (2005) ‘Molecular diagnosis and typing of Trypanosoma cruzi populations and lineages in cerebral chagas disease in a patient with AIDS’, *American Journal of Tropical Medicine and Hygiene*. American Society of Tropical Medicine and Hygiene, 73(6), pp. 1016–1018. doi: 10.4269/AJTMH.2005.73.1016.

**Burgos, J. M. *et al.*** (2008) ‘Case report: Molecular identification of Trypanosoma cruzi i tropism for central nervous system in chagas reactivation due to AIDS’, *American Journal of Tropical Medicine and Hygiene*. American Society of Tropical Medicine and Hygiene, 78(2), pp. 294–297. doi: 10.4269/AJTMH.2008.78.294.

**Camargos, S. *et al.*** (2017) ‘CNS chagoma’, *Neurology*. Wolters Kluwer Health, Inc. on behalf of the American Academy of Neurology, 88(6), pp. 605–606. doi: 10.1212/WNL.0000000000003600.

**Campo, M. *et al.*** (2010) ‘A woman with HIV infection, brain abscesses, and eosinophilia’, *Clinical Infectious Diseases*. Oxford Academic, 50(2), pp. 239–240. doi: 10.1086/649538/2/50-2-239-FIG002.GIF.

**Cardozo, A. *et al.*** (1992) ‘Meningoencefalitis chagásica en el síndrome de inmunodeficiencia adquirida (SIDA): primer caso en Uruguay’, *Rev. méd. Urug*, pp. 214–7.

**Del Castillo, M. *et al.*** (1990) ‘AIDS and Chagas’ disease with central nervous system tumor-like lesion’, *The American journal of medicine*. Am J Med, 88(6), pp. 693–694. doi: 10.1016/0002-9343(90)90544-N.

**Castillo, P. N. *et al.*** (2014) ‘Trypanosoma cruzi en paciente inmunocomprometido: Reactivación de enfermedad de Chagas en un sujeto residente en la ciudad de La Rioja, Argentina’, *Revista argentina de microbiología*. Asociación Argentina de Microbiología, 46(4), pp. 378–379. Available at: http://www.scielo.org.ar/scielo.php?script=sci_arttext&pid=S0325-75412014000500016&lng=es&nrm=iso&tlng=en.

**Castrillon, M. E.** (2013) ‘Enfermedad de Chagas cerebral en SIDA’, *Revista de la Facultad de Ciencias Médicas de Córdoba*, 70(4). doi: 10.31053/1853.0605.V70.N4.7405.

**Chalub, E. P. *et al.*** (2017) ‘Coinfección SIDA / Enfermedad de Chagas. Presentación de caso y revisión de la bibliografía’, *Prensa méd. argent*, pp. 489–494. Available at: https://prensamedica.com.ar/LPMA_V103_N09_comp.pdf.

**Choi, H. J. H. *et al.*** (2004) ‘Acute chagas’ disease presenting with a suprasellar mass and panhypopituitarism’, *Pituitary*. Pituitary, 7(2), pp. 111–114. doi: 10.1007/S11102-005-5350-4.

**Cicora, F. *et al.*** (2014) ‘Cerebral trypanosomiasis in a renal transplant recipient’, *Transplant Infectious Disease*. John Wiley & Sons, Ltd, 16(5), pp. 813–817. doi: 10.1111/TID.12265.

**Cohen, J. E. *et al.*** (1998) ‘Pseudotumoral chagasic meningoencephalitis as the first manifestation of acquired immunodeficiency syndrome’, *Surgical neurology*. Surg Neurol, 49(3), pp. 324–327. doi: 10.1016/S0090-3019(97)00173-0.

**Cohen, V. *et al.*** (2010) ‘Chagas’ disease as a cause of cerebral mass in a patient with lymphoblastic leukemia in remission’, *Archivos argentinos de pediatría*. Sociedad Argentina de Pediatría, 108(6), pp. 134–137. Available at: http://www.scielo.org.ar/scielo.php?script=sci_arttext&pid=S0325-00752010000600016&lng=en&nrm=iso&tlng=en.

**Cor José Antonio Frías Salcedo, T. M. *et al.*** (2006) ‘Cerebral trypanosomiasis in AIDS. Case report and literature review’, *Enfermedades Infecciosas y Microbiología*, 26(4), pp. 123–128.

**Cordova, E. *et al.*** (2008) ‘Reactivation of Chagas disease with central nervous system involvement in HIV-infected patients in Argentina, 1992-2007’, *International Journal of Infectious Diseases*. Elsevier, 12(6), pp. 587–592. doi: 10.1016/j.ijid.2007.12.007.

**Corti, M. *et al.*** (2000) ‘Chagas disease: another cause of cerebral mass occurring in patients with acquired immunodeficiency syndrome’, *Enfermedades Infecciosas y Microbiologia Clínica*, 18(4), pp. 194–195. Available at: 10932401.

**DiazGranados, C. A. *et al.*** (2009) ‘Chagasic encephalitis in HIV patients: common presentation of an evolving epidemiological and clinical association’, *The Lancet. Infectious diseases*. Lancet Infect Dis, 9(5), pp. 324–330. doi: 10.1016/S1473-3099(09)70088-X.

**Fernandes, H. J. *et al.*** (2017) ‘Case Report: Meningoencephalitis Caused by Reactivation of Chagas Disease in Patient Without Known Immunosuppression’, *The American Journal of Tropical Medicine and Hygiene*. The American Society of Tropical Medicine and Hygiene, 96(2), p. 292. doi: 10.4269/AJTMH.16-0225.

**Fernandez de Castro, A., Sanchez, A. and Cortes, N. L.** (2008) ‘Encefalitis chagásica: una complicación tardía postrasplante cardiaco’, *Universitas Medica*. Pontificia Universidad Javeriana, 49(1), pp. 112–120. Available at: https://www.redalyc.org/articulo.oa?id=231016462010.

**Fernández, M. L. *et al.*** (2021) ‘Benznidazole in Cerebrospinal Fluid: a Case Series of Chagas Disease Meningoencephalitis in HIV-Positive Patients’, *Antimicrobial Agents and Chemotherapy*. American Society for Microbiology (ASM), 65(3). doi: 10.1128/AAC.01922-20.

**Ferreira, M. S. *et al.*** (1997) ‘Reactivation of Chagas’ disease in patients with AIDS: report of three new cases and review of the literature’, *Clinical infectious diseases : an official publication of the Infectious Diseases Society of America*. Clin Infect Dis, 25(6), pp. 1397–1400. doi: 10.1086/516130.

**Fica Cubillos, A. *et al.*** (2017) ‘Chagas disease affecting the central nervous system in a patient with AIDS demonstrated by quantitative molecular methods’, *Revista chilena de infectología*. Sociedad Chilena de Infectología, 34(1), pp. 69–76. doi: 10.4067/S0716-10182017000100011.

**Gallo, P. *et al.*** (1992) ‘Acute central nervous system infection by Trypanosoma cruzi and AIDS’, *Arquivos de neuro-psiquiatria*. Arq Neuropsiquiatr, 50(3), pp. 375–377. doi: 10.1590/S0004-282X1992000300019.

**Gluckstein, D., Ciferri, F. and Ruskin, J.** (1992) ‘Chagas’ disease: another cause of cerebral mass in the acquired immunodeficiency syndrome’, *The American journal of medicine*. Am J Med, 92(4), pp. 429–432. doi: 10.1016/0002-9343(92)90275-G.

**Guidetto, B. *et al.*** (2019) ‘HIV and Chagas Disease Coinfection, a Tractable Disease?’, *Open Forum Infectious Diseases*. Oxford University Press, 6(7). doi: 10.1093/OFID/OFZ307.

**Hernández, C. *et al.*** (2014) ‘Chagas disease (Trypanosoma cruzi) and HIV co-infection in Colombia’, *International journal of infectious diseases : IJID : official publication of the International Society for Infectious Diseases*. Int J Infect Dis, 26, pp. 146–148. doi: 10.1016/J.IJID.2014.04.002.

**Kaushal, M. *et al.*** (2019) ‘Cerebral Trypanosomiasis in an Immunocompromised Patient: Case Report and Review of the Literature’, *World Neurosurgery*. Elsevier, 129, pp. 225–231. doi: 10.1016/J.WNEU.2019.05.260.

**Koff, A. *et al.*** (2020) ‘Under Our Very Eyes’, *The New England journal of medicine*. N Engl J Med, 382(10), pp. 952–957. doi: 10.1056/NEJMCPS1902835.

**Lages-Silva, E. *et al.*** (2002) ‘Chagasic meningoencephalitis in a patient with acquired immunodeficiency syndrome: diagnosis, follow-up, and genetic characterization of Trypanosoma cruzi’, *Clinical infectious diseases : an official publication of the Infectious Diseases Society of America*. Clin Infect Dis, 34(1), pp. 118–123. doi: 10.1086/324355.

**Lambert, N. *et al.*** (2006) ‘Chagasic encephalitis as the initial manifestation of AIDS’, *Annals of internal medicine*. Ann Intern Med, 144(12), pp. 941–943. doi: 10.7326/0003-4819-144-12-200606200-00018.

**Lopez-Albizu, C. *et al.*** (2021) ‘Case report of Chagas disease reactivation: new diagnosis tool by direct microscopic observation of biopsy specimen and its preservation fluid’, *Revista da Sociedade Brasileira de Medicina Tropical*. Brazilian Society of Tropical Medicine, 54, pp. 1–4. doi: 10.1590/0037-8682-0326-2020.

**Di Lorenzo, G. A. *et al.*** (1996) ‘Chagasic granulomatous encephalitis in immunosuppressed patients. Computed tomography and magnetic resonance imaging findings’, *Journal of neuroimaging : official journal of the American Society of Neuroimaging*. J Neuroimaging, 6(2), pp. 94–97. doi: 10.1111/JON19966294.

**Lury, K. M. and Castillo, M.** (2005) ‘Chagas’ disease involving the brain and spinal cord: MRI findings’, *AJR. American journal of roentgenology*. AJR Am J Roentgenol, 185(2), pp. 550–552. doi: 10.2214/AJR.185.2.01850550.

**Madalosso, G. *et al.*** (2004) ‘Chagasic meningoencephalitis: case report of a recently included AIDS-defining illness in Brazil’, *Revista do Instituto de Medicina Tropical de Sao Paulo*. Rev Inst Med Trop Sao Paulo, 46(4), pp. 199–202. doi: 10.1590/S0036-46652004000400005.

**Marchiori, P. E. *et al.*** (2007) ‘Late reactivation of Chagas’ disease presenting in a recipient as an expansive mass lesion in the brain after heart transplantation of chagasic myocardiopathy’, *The Journal of heart and lung transplantation : the official publication of the International Society for Heart Transplantation*. J Heart Lung Transplant, 26(11), pp. 1091–1096. doi: 10.1016/J.HEALUN.2007.07.043.

**Marques de Brito, C. M., Pires, M. Q. and Pacheco, R. S.** (2003) ‘Chagas disease and HIV co-infection: genetic analyses of two Trypanosoma cruzi strains under experimental immunosuppression’, *Kinetoplastid Biology and Disease*. BMC, 2, p. 17. doi: 10.1186/1475-9292-2-17.

**De Medeiros, M. B., Guerra, J. A. D. O. and De Lacerda, M. V. G.** (2008) ‘Meningoencephalitis in a patient with acute Chagas disease in the Brazilian Amazon’, *Revista da Sociedade Brasileira de Medicina Tropical*. Sociedade Brasileira de Medicina Tropical - SBMT, 41(5), pp. 520–521. doi: 10.1590/S0037-86822008000500020.

**Menghi, C. I., Gatta, C. L. and Arcavi, M.** (2010) ‘[Trypanosoma cruzi in the cerebrospinal fluid of an AIDS patient]’, *Revista Argentina de microbiologia*. Rev Argent Microbiol, 42(2), p. 142. doi: 10.1590/S0325-75412010000200017.

**Montero, A. *et al.*** (1998) ‘Empirical anti-toxoplasma therapy in cerebral AIDS and Chagas disease. Presentation of 2 cases, review of the literature and proposal of an algorithm’, *Medicina [B.Aires]*, 58, pp. 504–506.

**Montero, M. *et al.*** (2019) ‘High-dose benznidazole in a 62-year-old Bolivian kidney transplant recipient with Chagas central nervous system involvement’, *International journal of infectious diseases : IJID : official publication of the International Society for Infectious Diseases*. Int J Infect Dis, 78, pp. 103–106. doi: 10.1016/J.IJID.2018.10.014.

**Moretta, G. *et al.*** (2009) ‘Clinical Reasoning: A 35-year-old man with a right hemiplegia and a cerebral mass’, *Neurology*. Wolters Kluwer Health, Inc. on behalf of the American Academy of Neurology, 73(8), pp. e35–e38. doi: 10.1212/WNL.0B013E3181B56D9E.

**Multani, A. *et al.*** (2019) ‘Diagnosis of Chagasic Encephalitis by Sequencing of 28S rRNA Gene’, *Emerging Infectious Diseases*. Centers for Disease Control and Prevention, 25(7), p. 1370. doi: 10.3201/EID2507.180285.

**Nijjar, S. S. and Del Bigio, M. R.** (2007) ‘Clinical Vistas: Cerebral trypanosomiasis in an incarcerated man’, *CMAJ : Canadian Medical Association Journal*. Canadian Medical Association, 176(4), p. 448. doi: 10.1503/CMAJ.060540.

**Nishioka, S. A. *et al.*** (1993) ‘Reactivation of Chagas’ disease successfully treated with benznidazole in a patient with acquired immunodeficiency syndrome’, *Memorias do Instituto Oswaldo Cruz*. Mem Inst Oswaldo Cruz, 88(3), pp. 493–496. doi: 10.1590/S0074-02761993000300022.

**Oddó, D. *et al.*** (1992) ‘Acute Chagas’ disease (Trypanosomiasis americana) in acquired immunodeficiency syndrome: report of two cases’, *Human pathology*. Hum Pathol, 23(1), pp. 41–44. doi: 10.1016/0046-8177(92)90009-R.

**Olga, L. M.** (2010) ‘Three-year survival of a patient with HIV and chagasic meningoencephalitis: Case report’, *Revista chilena de infectología*. Sociedad Chilena de Infectología, 27(2), pp. 160–164. doi: 10.4067/S0716-10182010000200012.

**de Oliveira Santos, E. *et al.*** (2002) ‘Reactivation of Chagas’ disease leading to the diagnosis of acquired immunodeficiency syndrome’, *The Brazilian journal of infectious diseases : an official publication of the Brazilian Society of Infectious Diseases*. Braz J Infect Dis, 6(6), pp. 317–321. doi: 10.1590/S1413-86702002000600009.

**Pacheco, R. S. *et al.*** (1998) ‘Chagas’ disease and HIV co-infection: genotypic characterization of the Trypanosoma cruzi strain’, *Memorias do Instituto Oswaldo Cruz*. Mem Inst Oswaldo Cruz, 93(2), pp. 165–169. doi: 10.1590/S0074-02761998000200005.

**Pagano, M. A. *et al.*** (1999) ‘Cerebral tumor-like American trypanosomiasis in acquired immunodeficiency syndrome’, *Annals of Neurology*. Available at: https://www.academia.edu/22551296/Cerebral_tumor_like_American_trypanosomiasis_in_acquired_immunodeficiency_syndrome.

**Pimentel, P. C. A., Handfas, B. W. and Carmignani, M.** (1996) ‘Trypanosoma cruzi meningoencephalitis in AIDS mimicking cerebral metastases: case report’, *Arquivos de neuro-psiquiatria*. Arq Neuropsiquiatr, 54(1), pp. 102–106. doi: 10.1590/S0004-282X1996000100017.

**Reimer-McAtee, M. J. *et al.*** (2021) ‘HIV and Chagas Disease: An Evaluation of the Use of Real-Time Quantitative Polymerase Chain Reaction to Measure Levels of Trypanosoma cruzi Parasitemia in HIV Patients in Cochabamba, Bolivia’, *The American Journal of Tropical Medicine and Hygiene*. The American Society of Tropical Medicine and Hygiene, 105(3), p. 643. doi: 10.4269/AJTMH.20-1141.

**Rocha, A. *et al.*** (1993) ‘Trypanosoma cruzi meningoencephalitis and myocarditis in a patient with acquired immunodeficiency syndrome’, *Revista do Instituto de Medicina Tropical de Sao Paulo*. Rev Inst Med Trop Sao Paulo, 35(2), pp. 205–208. doi: 10.1590/S0036-46651993000200014.

**Salgado, P. R. *et al.*** (1996) ‘Tumor-like lesion due to Chagas’ disease in a patient with lymphocytic leukemia’, *Revista do Instituto de Medicina Tropical de Sao Paulo*. Rev Inst Med Trop Sao Paulo, 38(4), pp. 285–288. doi: 10.1590/S0036-46651996000400008.

**Sartori, A. M. C. *et al.*** (2007) ‘Manifestations of Chagas disease (American trypanosomiasis) in patients with HIV/AID S’, *Annals of Tropical Medicine and Parasitology*. Maney Publishing, 101(1), pp. 31–50. doi: 10.1179/136485907X154629.

**Sica, R. E. P., Gargiullo, G. and Papayanis, C.** (2008) ‘Tumour-like chagasic encephalitis in AIDS patients: an atypical presentation in one of them and outcome in a small series of cases’, *Arquivos de neuro-psiquiatria*. Arq Neuropsiquiatr, 66(4), pp. 881–884. doi: 10.1590/S0004-282X2008000600021.

**Silva, N. *et al.*** (1999) ‘Trypanosoma cruzi meningoencephalitis in HIV-infected patients’, *Journal of acquired immune deficiency syndromes and human retrovirology : official publication of the International Retrovirology Association*. J Acquir Immune Defic Syndr Hum Retrovirol, 20(4), pp. 342–349. doi: 10.1097/00042560-199904010-00004.

**Simioli, F. *et al.*** (2017) ‘Chagas disease in the central nervous system in patient infected with HIV: diagnostic and therapeutic difficulties’, *Revista chilena de infectología*. Sociedad Chilena de Infectología, 34(1), pp. 62–66. doi: 10.4067/S0716-10182017000100009.

**Solari, A. *et al.*** (1993) ‘Successful treatment of Trypanosoma cruzi encephalitis in a patient with hemophilia and AIDS’, *Clinical infectious diseases : an official publication of the Infectious Diseases Society of America*. Clin Infect Dis, 16(2), pp. 255–259. doi: 10.1093/CLIND/16.2.255.

**De Sousa Dos Santos, S. *et al.*** (1999) ‘Ocular myositis and diffuse meningoencephalitis from Trypanosoma cruzi in an AIDS patient’, *Transactions of the Royal Society of Tropical Medicine and Hygiene*. Trans R Soc Trop Med Hyg, 93(5), pp. 535–536. doi: 10.1016/S0035-9203(99)90370-X.

**Spadafora, M. S. R. *et al.*** (2014) ‘Trypanosoma cruzi Necrotizing Meningoencephalitis in a Venezuelan HIV+-AIDS Patient: Pathological Diagnosis Confirmed by PCR Using Formalin-Fixed- and Paraffin-Embedded-Tissues’, *Analytical Cellular Pathology (Amsterdam)*. Hindawi Limited, 2014. doi: 10.1155/2014/124795.

**Sztokhamer, D. *et al.*** (2010) ‘Reactivación de enfermedad de Chagas (Tripanosomiasis americana) con compromiso cerebral, en pacientes VIH/SIDA en Argentina’, *Actual. SIDA*, pp. 49–54.

**Valerga, M. *et al.*** (2005) ‘[Multifocal encephalitis in an AIDS patient]’, *Enfermedades infecciosas y microbiologia clinica*. Enferm Infecc Microbiol Clin, 23(9), pp. 569–570. doi: 10.1157/13080268.

**Verdú, J. *et al.*** (2009) ‘Reactivation of Chagas disease with central nervous system involvement: peripheral blood smear evidence’, *International journal of infectious diseases : IJID : official publication of the International Society for Infectious Diseases*. Int J Infect Dis, 13(6). doi: 10.1016/J.IJID.2009.02.013.

**Yasukawa, K. *et al.*** (2014) ‘Trypanosoma cruzi meningoencephalitis in a patient with acquired immunodeficiency syndrome’, *The American journal of tropical medicine and hygiene*. Am J Trop Med Hyg, 91(1), pp. 84–85. doi: 10.4269/AJTMH.14-0058.

**Yoo, T. W. *et al.*** (2004) ‘Concurrent cerebral american trypanosomiasis and toxoplasmosis in a patient with AIDS’, *Clinical infectious diseases : an official publication of the Infectious Diseases Society of America*. Clin Infect Dis, 39(4). doi: 10.1086/422456.
